# Add-On Effect of Chinese Herbal Medicine Bath to Phototherapy for Psoriasis Vulgaris: A Systematic Review

**DOI:** 10.1155/2013/673078

**Published:** 2013-07-25

**Authors:** Jason Jingjie Yu, Claire Shuiqing Zhang, Anthony Lin Zhang, Brian May, Charlie Changli Xue, Chuanjian Lu

**Affiliations:** ^1^Department of Dermatology, The Second Clinical College, Guangzhou University of Chinese Medicine, Guangzhou 510120, China; ^2^Traditional & Complementary Medicine Research Program, Health Innovations Research Institute, School of Health Sciences, RMIT University, Bundoora Campus, Melbourne, VIC 3083, Australia; ^3^Guangdong Provincial Academy of Chinese Medical Sciences and Guangdong Provincial Hospital of Chinese Medicine, Guangzhou 510120, China

## Abstract

Psoriasis vulgaris is the most common form of psoriasis. Phototherapy has been proven effective for psoriasis, but side effects have become a concern. Chinese herbal medicine (CHM) bath combined with phototherapy has been used in clinical settings, but the additional benefit requires evaluation. This review aims to evaluate the additional benefit and safety of adding CHM bath to phototherapy for psoriasis vulgaris. Cochrane library, PubMed, Embase, CNKI, and CQVIP were searched from their inceptions to 6 August 2012. Randomized controlled trials (RCTs) comparing CHM bath plus phototherapy to phototherapy alone for psoriasis vulgaris were included. Data was analyzed using Review Manager 5.1.0. Thirteen RCTs were included in the review, and eight were included in the meta-analysis. Meta-analysis showed higher efficacy of CHM bath plus phototherapy when compared with phototherapy alone in terms of PASI 60 (RR 1.25; 95% CI: 1.18–1.32). Mild adverse events were reported in ten studies, but these could be alleviated by reducing UV dosage or applying emollient. In conclusion, CHM bath appears to be a beneficial and safe adjunctive therapy to phototherapy for psoriasis vulgaris. However, these results should be interpreted with caution due to the low methodological quality of the included studies.

## 1. Introduction

Psoriasis is a chronic, inflammatory, and systemic disorder that is characterized by scaling and erythematous plaques, which may be severely pruritic or painful. The prevalence of psoriasis varies in different regions of the world, with an average prevalence of approximately 2% in western industrialized countries [1, 2]. Psoriasis is associated with metabolic syndrome and several inflammatory diseases including rheumatoid arthritis and Crohn's disease [3]. For most patients, psoriasis brings varying degrees of lifelong restriction, not only from the skin lesions but also from joint pain and stiffness in some patients with arthritic type psoriasis and even severe discrimination in some cases [4]. In addition, psoriasis causes significant economic burden in health care costs and losses in productivity [4].

Psoriasis vulgaris (or plaque psoriasis) is the most common clinical manifestation of psoriasis, affecting approximately 80% to 90% of psoriasis patients [1, 4], with its main characteristic being erythematosquamous plaques at certain sites [1, 4]. Inappropriate medication or infection may cause psoriasis vulgaris to transfer to other types of psoriasis, including erythrodermic or pustular types [1, 4]. 

Phototherapy involves exposure of patients to specific wavelengths of light, either ultraviolet A (UVA) or ultraviolet B (UVB) [1, 4]. It has been widely used as an effective therapeutic approach for psoriasis and is considered free of the side effects associated with conventional systemic therapies [1, 4]. However, some undesirable side effects can be induced by phototherapy, including erythema and pruritus [18–21]. Long-term use of phototherapy especially the combination of oral methoxsalen (psoralen) and ultraviolet A radiation (PUVA) therapy [4, 22] increases the risk of developing skin cancer. Chinese herbal medicine (CHM) bath combined with phototherapy have been widely used in clinical practice for patients with psoriasis. It has been reported that the combination of CHM bath with phototherapy has superior efficacy [5, 6] and fewer side effects, and can reduce ultraviolet (UV) dosage in comparison with phototherapy alone [5]. However, a systematic review following rigorous methodology to evaluate the evidence is lacking. Therefore, this study aims to evaluate the efficacy and safety of CHM bath combined with phototherapy for psoriasis vulgaris.

## 2. Methods

### 2.1. Search Methods

The Cochrane library, PubMed, Embase, China National Knowledge Infrastructure (CNKI), and Chinese Scientific Journals Full Text Database (CQVIP) were searched from their respective inceptions to 6 August 2012. Search terms were in three groups: clinical condition (psoriasis, etc.), intervention (herbal medicine bath, phototherapy, etc.) and study type (controlled trial, etc.), with adjustments for different databases. The reference lists of retrieved review articles were searched for additional studies.

### 2.2. Study Selection Criteria

Randomized controlled trials (RCTs) published in English or Chinese, regardless of publication type, that compared CHM bath plus phototherapy with phototherapy alone for psoriasis vulgaris, using symptom scoring systems to evaluate the efficacy, were considered. The phototherapy was defined as UVA and/or UVB. Co-interventions were allowed as long as the same intervention was administered in both arms of the study. Studies of other types of psoriasis using other forms of CHM were excluded. 

### 2.3. Data Extraction

Data were extracted into a predefined form in Excel and categorized by two authors (Jason Jingjie Yu and Claire Shuiqing Zhang).

### 2.4. Risk of Bias Assessment

Two authors (Jason Jingjie Yu and Claire Shuiqing Zhang) assessed the studies using the Cochrane collaboration's tool for assessing risk of bias [23]. Any disagreement was resolved through discussion with a third author (Lin Zhang).

### 2.5. Meta-Analysis

Studies with consistent interventions, outcome measures, and sufficient data were pooled in the meta-analysis using the Cochrane Review Manager 5.1 (RevMan5.1) [23]. Dichotomous data was assessed using risk ratio (RR) with a 95% confidence interval (CI) [23]. A fixed-effect or random-effect model was used according to heterogeneity [23].

## 3. Results

**Figure 1:**
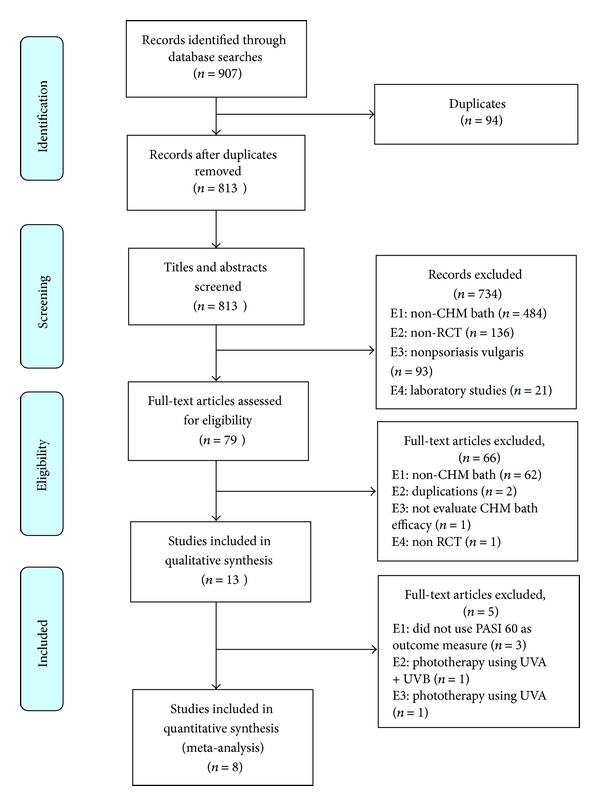
study selection PRISMA flow chart. CHM: Chinese herbal medicine; RCT: randomized controlled trial. PASI: the psoriasis area and severity index; UVA/UVB: ultraviolet A/B.

The searches yielded 907 relevant articles. After removing duplicates, 813 records remained. 79 full articles were retrieved for further evaluation after screening titles and abstracts. Thirteen studies satisfied all selection criteria and were included in this review. All were published in Chinese. Eight studies were included in the meta-analysis (Figure 1). 

### 3.1. Description of Studies

The characteristics of included studies are summarized in Table 1.

#### 3.1.1. Participants

All included RCTs were conducted in hospitals in China; a total of 2168 outpatient participants were involved in the 13 studies, whilst there were 1226 participants in the eight studies included in the meta-analysis. The average ages of the participants ranged from 27.4 to 45.8 years. Ten studies specified the western medicine (WM) diagnostic criteria used for patient recruitment [5, 6, 8–10, 12–15, 17] and four studies employed Chinese medicine (CM) syndrome differentiation criteria [5, 9, 14, 15]. The key points of the diagnostic criteria referred to in these studies were consistent with “Guidelines on the treatment of psoriasis vulgaris” [4] or “Consensus of Diagnosis and Treatment of Psoriasis Vulgaris in Integrative Medicine (China)” [24]. The disease duration ranged from one to 13 years. Four studies recruited patients with psoriasis vulgaris at the stable stage [5, 6, 8, 17], while the other studies did not provide such information. None of the studies used symptom severity as their inclusion criteria. All studies claimed balanced baseline data. Psoriasis Area and Severity Index (PASI) scores were reported in four studies and the average PASI ranged from 10.9 to 40.2 at baseline [5, 7, 14, 15]. Dermatology Life Quality Index (DLQI) or Body Surface Area (BSA) data were not measured in any of the studies.

#### 3.1.2. Interventions

In regard to the phototherapy, eleven studies employed narrowband (NB)-UVB [5–8, 10–16], one study used a combination of UVA and UVB [9], and one study used UVA [17]. None used PUVA. The dosages of phototherapy varied across studies. Six studies used NB-UVB (310 to 315 nm) with 0.2 to 0.4 J/cm^2^ as the initial dosage and then increased the dose by 0.1 J/cm^2^ each time [6, 7, 10, 12, 13, 15]. Phototherapy was usually 2 to 3 sessions a week and the total duration was 21 to 90 days (Table 1).

The CHM formula used for the bath varied across the 13 studies (Table 2). The most frequently used herbs were *Salvia miltiorrhiza *root (Dan shen) [6, 8–10, 12, 13, 16], *Dictamnus dasycarpus *bark (Bai xian pi) [6, 7, 9, 10, 12, 13, 16],* Sophora flavescens *root (Ku shen) [7, 8, 10, 11, 14, 16, 17], and *Kochia scoparia *fruit (Di fu zi) [6, 7, 10–13, 17]. The CHM bath was conducted before phototherapy for 20 to 30 minutes in each treatment session. 

Co-interventions were used in both arms of five studies, they were: (1) *PuLian* ointment—*Scutellaria baicalensis *root (Huang qin), *Phellodendron amurense *bark (Huang bo), and petroleum jelly [6]; (2) *Bing Huang Fu Le* ointment—*Rheum palmatum *root (Da huang)*, Curcuma longa *rhizome (Jiang huang)*, Scutellaria baicalensis* root (Huang qin)*, Glycyrrhiza uralensis *root (Gan cao), sulphur (Liu huang), synthetic borneol (Bing pian), and menthol (Bo he nao) [7]; (3) urea emollient (produced by the hospital) which was used as moisturizer in three studies [7, 14, 24]; and (4) glycyrrhizin tablets [24]. In addition, two studies conducted one-week pretrial treatments as co-interventions in both arms using acitretin capsules [11] or halometasone emollient [14]. 

#### 3.1.3. Outcome Measures

The primary outcome measure in all 13 studies was “total effective rate” (TER) which was defined as PASI score reduction in ten studies [5–10, 12, 14, 15, 17], lesion improvement based on other criteria in two studies [11, 16], and one study did not specify the criteria used [13]. Actual PASI score was also reported in four studies [5, 7, 14, 15]. Based on a European consensus on treatment goals for moderate to severe psoriasis, PASI 50 is the minimum requirement for the efficacy of any therapy for psoriasis, but these criteria were not followed in any of the included studies [25].

In general, the effectiveness of the interventions was reported in four levels: “clinically cured”, “remarkably effective”, “effective”, and “ineffective”, according to the reduction of PASI or symptom scores in the included studies except for one study that used three levels (clinically cured, remarkably effective, and ineffective) [16]. However, the definition of these levels varied across studies. For example, in studies using PASI score as the measurement [5–10, 12, 14, 15, 17], the levels of effectiveness were defined as PASI (95-60-20) [14], PASI (90-60-25) [5–9, 17], or PASI (90-60-30) [15]. For PASI (95-60-20), “clinically cured” was defined as a PASI score reduction of 95 to 100%; “remarkably effective” was a reduction of 60 to 94%; “effective” was a reduction of 20 to 59%; and “ineffective” was a reduction of 0 to 19% [14]. Since PASI 60 was used in ten studies [5–10, 12, 14, 15, 17] as the benchmark of “remarkably effective” for TER calculation, it was selected to be the outcome for pooling studies for meta-analysis in this review. Three studies were excluded from meta-analysis because two of them used TER based on a scoring system other than PASI, for which there was no evidence supporting it as an appropriate approach to evaluate therapeutic effect [11, 16]; and one was excluded due to lack of a clear description of how “lesion improvement” was measured [13].

#### 3.1.4. Efficacy

All thirteen studies reported that the combination of CHM bath and phototherapy had a superior efficacy compared to phototherapy alone in terms of TER. In addition, Cui et al., 2008 reported that the administration of CHM bath could significantly reduce NB-UVB average dosage [5].

#### 3.1.5. Followup

Followup and relapse rate were reported in two studies [6, 9]. In one, the relapse rate of the treatment group was 15.2% compared with 35.2% for control [9]. In the other, the rates were 10.8% for the treatment group and 30.3% in the control. In both cases, a significant difference between the two groups was reported (*P* < 0.05) but a definition of “relapse” was not provided in either study.

#### 3.1.6. Dropouts

One study reported the number of dropouts during the treatment period without detailed reasons [6]. All other studies reported that the number of patients who completed the trial was same as the number randomized.

#### 3.1.7. Adverse Events (AEs)

Three studies did not report any AEs [8, 11, 17]. The most frequent AEs reported by the other ten studies were pruritus (7 studies), skin dryness (6 studies), redness (6 studies), burning pain (3 studies), and skin pigmentation (3 studies). Three studies [5–7] reported significantly higher rates of redness and pruritus in the UVB control groups. One of these three studies also reported no significant difference between groups for skin dryness [7]. The other seven studies that reported on AEs did not provide details of between-group comparisons of AEs. All AEs reported in the ten studies could be resolved by reducing UV dosage or applying emollient. No serious adverse event (SAE) was reported.

### 3.2. Risks of Bias Assessment

**Figure 2:**
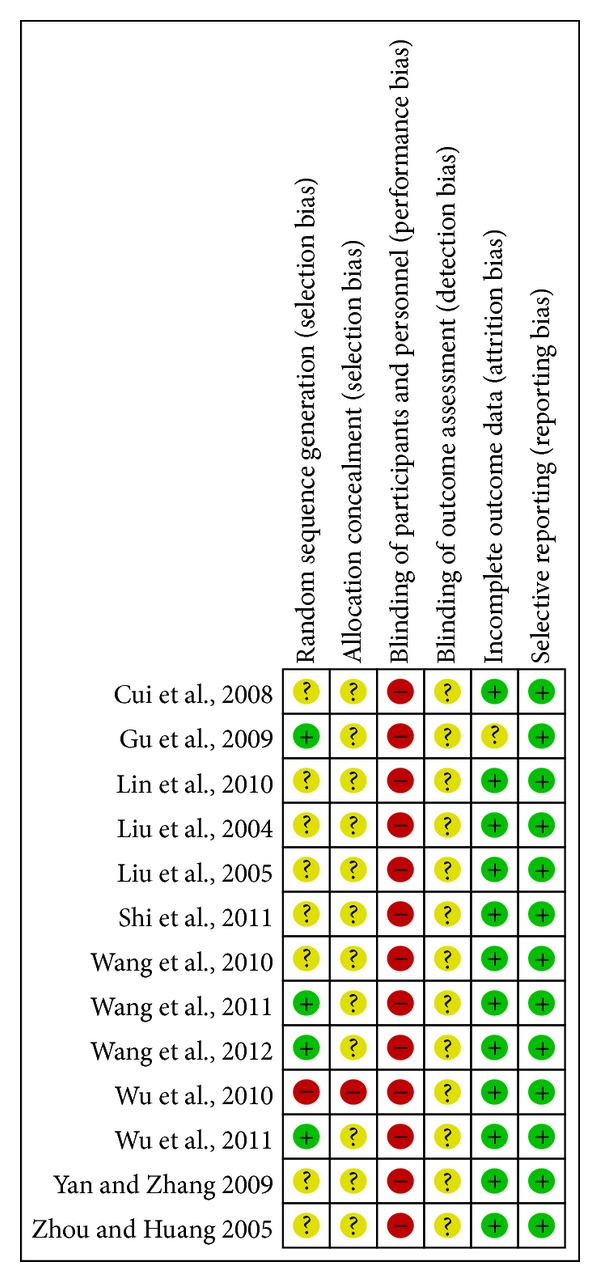
Risk of bias summary.

Risk of bias was assessed following the Cochrane Handbook 5.1.0 [23] and is summarized in Figure 2. Although all thirteen studies claimed “randomized,” only six studies described the specific randomization methods [5, 6, 10, 12–14]. Among them, the random sequence generation in four studies was appropriate [6, 12–14] and assessed as “low risk”; one study was assessed as “high risk” due to the use of patients' visiting order [15]; one study used “envelopes” for randomization, which is not an adequate description to judge bias; therefore, it was assessed as “unclear” [5]. The other seven studies were considered “unclear” due to the lack of information about randomization [7–11, 16, 17]. For allocation concealment method, the study that used patients' visiting order for group allocation was judged “high risk” [15] while the other twelve studies did not provide adequate information and therefore obtained judgments of “unclear.” For all thirteen studies, no information was provided for blinding of participants, personnel, or outcome assessors. In fact, blinding was impossible for participants and personnel in these studies due to the design. One study reported the number of dropouts without reasons; hence, it was judged as “unclear” for incomplete outcome data [6]; the other twelve studies were assessed as “low risk” because they reported that all participants finished the study. A “low risk” assessment was given to all studies in terms of “selective outcome reporting” as the outcomes reported in the results section matched the methods section. However, none had a protocol registered. 

### 3.3. Meta-Analysis

Two studies were not pooled in the meta-analysis since they used different forms of phototherapy compared to the others: one combined UVA and UVB [9] and the other used UVA [17]. Three studies were not included in the meta-analysis because they did not employ PASI 60 [11, 13, 16]. As a result, eight RCTs which used NB-UVB as phototherapy and PASI 60 for TER calculation were pooled in the meta-analysis. These were divided into two groups based on study design as follows.

**Figure 3:**
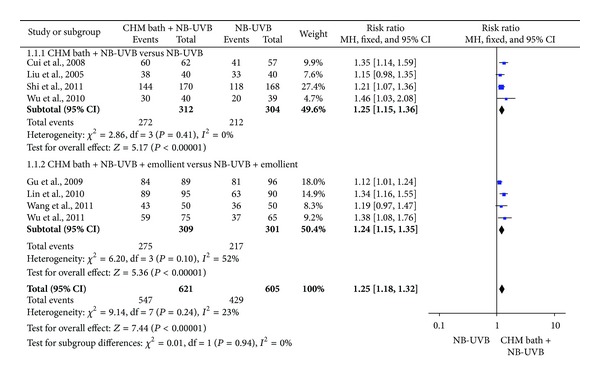
Forest plot of clinical efficacy (PASI 60). PASI: the psoriasis area and severity index. PASI 60: the percentage of participants who achieved PASI scores reduced by ≥60%. CHM: Chinese herbal medicine. NB-UVB: narrowband ultraviolet.

*Group 1: CHM Bath + NB-UVB versus NB-UVB.* Four studies [5, 8, 10, 15] showed a higher TER (PASI 60) for CHM bath plus phototherapy compared with phototherapy alone (RR 1.25; 95% CI: 1.15–1.36) without heterogeneity (*I* ^2^ = 0%) (Figure 3). There was diversity in the herbs used in these studies but two studies had two main herbs, Ku shen and Dan shen, in common [8, 10]. The pooled result for these studies favoured the addition of the herbal bath (RR 1.19; 95% CI: 1.08–1.32, *I* ^2^ = 0%).

*Group 2: CHM Bath + NB-UVB + Emollient versus NB-UVB + Emollient.* Four studies [6, 7, 12, 14] showed a higher TER (PASI 60) for CHM bath plus phototherapy plus externally applied emollient cream, compared with phototherapy plus the same cream (RR 1.24; 95% CI: 1.15–1.35). The heterogeneity was moderate (*I* ^2^ = 52%) (Figure 3). Of the four studies, three had main herbs in common [6, 7, 12] and of these Gu et al. 2009 and Wang et al. 2011 studies [6, 12] were the most similar in that they shared Dan shen and Bai xian pi. When the dissimilar study [12] was excluded, there was little change in the result (RR 1.21; 95% CI: 1.12–1.32) and the heterogeneity remained moderate (*I* ^2^ = 54%) but when the pooling was limited to the two studies with the most similar CHMs (Gu et al. 2009 and Wang et al. 2011) [6, 12] the heterogeneity was removed (RR 1.14; 95% CI: 1.04–1.26, *I* ^2^ = 0%).

Overall, the above eight studies [5–8, 10, 12, 14, 15] had a higher TER (PASI 60) for CHM bath plus phototherapy, compared with the phototherapy (RR 1.25; 95% CI: 1.18–1.32). The heterogeneity was relatively low (*I* ^2^ = 23%) (Figure 3).

## 4. Discussion

### 4.1. Efficacy of Phototherapy Combined with CHM Bath for Psoriasis

All thirteen studies reported that CHM bath plus phototherapy had superior efficacy to phototherapy alone. In the meta-analysis for group 1 (CHM bath + NB-UVB versus NB-UVB), all studies used the same primary outcome measure, did not use other supplementary treatment, and there was no heterogeneity, so this result can be considered the most robust. Nevertheless, none of the studies employed an identical herbal bath formulation although there were herbal ingredients in common. In contrast, there was considerable variation in study design in group 2 (CHM bath + NB-UVB + emollient versus NB-UVB + emollient) and this was reflected in the heterogeneity found in the meta-analysis results. In fact, some co-intervention medications used in group 2, such as *PuLian* ointment and *Bing Huang Fu Le *ointment, have been reported to have therapeutic effects on psoriasis [26, 27]. Whether there was any interaction between the interventions and co-interventions was unclear. 

### 4.2. Possible Mechanisms of Action for Phototherapy Combined with CHM Bath

In psoriasis treatment, phototherapy mainly produces local immunosuppressive effects and anti-proliferative effects [1, 4]. The primary immunosuppressive actions are reducing mobility of antigen-presenting Langerhans cells and inhibiting T lymphocyte activation [1, 4]. In addition, phototherapy inhibits epidermal hyperproliferation by interactions with keratinocyte DNA [1, 4]. It has been reported that more than 50% of patients treated by phototherapy achieved at least 75% improvement in PASI score (PASI 75) after four to six weeks based on the recommendations of the latest treatment guideline for psoriasis vulgaris [4]. In this review, for the ten studies [5–10, 12, 14, 15, 17] that used PASI 60 for calculating TER, more than 50% of control group patients (611 out of 913) achieved PASI 60 after four to eight weeks treatment with phototherapy. Although the proportion of control group patients who achieved PASI 75 was not specifically assessed, this result indicates that the phototherapy was effective.

The additional therapeutic effects of the CHM bath could be attributable to three main aspects: (1) the thermotherapy effects; (2) the effects of a water bath; and (3) the specific therapeutic effects of the CHMs used in the baths. Each of these aspects is discussed below.

#### 4.2.1. Thermal Effects

Hyperthermia is not a new concept but research interest into its application in the treatment of psoriasis is relatively recent [28]. Early utilization of hyperthermia for psoriasis followed on from the ideas that the severity of psoriasis would reduce during warm summer months, and a benefit of hyperthermia had been shown for killing cancer cells [29]. Orenberg's study in 1980, which used ultrasound induced heat to treat psoriasis, showed a benefit for psoriasis lesion remission [29]. Later it was demonstrated that hyperthermia induced by topical exothermic pads [30], infrared [31], or microwave [32] was also effective for psoriasis plaques. The advantage of using a hot water bath is that it is a simple, inexpensive approach that can be used to treat relatively large body areas [33]. 

#### 4.2.2. Water Baths (Balneotherapy)

Since hyperthermia has proven beneficial for psoriasis lesions, the simple modality of using warm or hot water became popular. Nowadays, various types of water bath therapy are considered safe treatments for dermatological conditions and are practiced in many countries. Well-known balneotherapy sites include the Dead Sea in Israel, the Kangal hot spring in Turkey, the Kusatsu hot spring in Japan, and the Blue Lagoon in Iceland. In particular, the Dead Sea is known to be effective for the treatment of psoriasis [34]. The mechanisms by which diseases respond to balneotherapy probably incorporate chemical, thermal and mechanical effects [35]. It was suggested by a clinical study that simple repetitive water bath hyperthermia was effective in the treatment of psoriasis by improving psoriatic lesions, reducing edema and relieving pruritus [28]. In addition, the water itself could remove psoriatic scale [36], which may be beneficial for skin absorption of phototherapy. Previous studies have shown that water bath markedly increased photosensitivity to UVB [37, 38]. 

#### 4.2.3. Specific Effects of the CHMs Used in the Baths

Only one of the herbs used in one study, *Psoralea corylifolia* (Bu gu zhi) [5], is known to increase photosensitivity; therefore, the overall effectiveness reported for the CHM baths does not appear to be due to pharmacological enhancement of the effects of the phototherapy.

In order to investigate the plausibility of the CHMs used in the baths having antipsoriatic actions, experimental reports on the actions of the main herbal ingredients were examined. The most frequently used herbs in the included studies were *Salvia miltiorrhiza *root (Dan shen) [6, 8, 9, 12, 13, 16, 23], *Dictamnus dasycarpus *bark (Bai xian pi) [6, 7, 9, 10, 12, 13, 16], *Sophora flavescens *root (Ku shen) [7, 8, 10, 11, 14, 16, 17], and *Kochia scoparia *fruit (Di fu zi) [6, 7, 10–13, 17] which were each used in seven studies. Each of these herbs has a history of topical application in the management of dermatological disorders [39]. Also, experimental studies have been conducted on extracts and/or compounds derived from these herbs with regard to actions relevant to psoriasis. 

*Salvia miltiorrhiza *(Dan shen) root and its constituent tanshinones and salvianolic acids have received considerable research attention for the treatment and prevention of cardiovascular disorders [40]. These compounds have been shown to have free radical scavenging and anti-inflammatory effects [40–42]. Also, a number of its constituent tanshinones have been reported to have antiproliferative or proapoptotic effects in cancer cell lines [43]. More specifically, an *in vitro *study of mouse keratinocytes indicated that tanshinone IIA time and dose dependently inhibited cell growth by inducing apoptosis via caspase cascade [44]. Another possible mechanism of action for tanshinone IIA in keratinocytes is via inhibiting the dimerization of the activator protein 1 (AP-1) transcription factor resulting in reduced interferon sensitivity which in turn could lead to a reduced inflammatory response [45]. 

Jiang et al., 2008 [46] reported that an extract of the root bark of *Dictamnus dasycarpus *(Bai xian pi) inhibited histamine release and reduced scratching behaviour in a mouse anaphylaxis model. *In vitro* anti-inflammatory activity has been reported for a root-bark extract [47] and for its constituents fraxinellone [48] and obacunone [49]. Also, its essential oil and a number of compounds including dictamnine, preskimmianine, and fraxinellone have demonstrated antitumour activity [47]. 

In the case of *Sophora flavescens *(Ku shen) root, an extract inhibited histamine release both *in vitro* and *in vivo* [50, 51], and other experimental studies have reported anti-inflammatory effects for its constituent alkaloids matrine [52, 53] and oxymatrine [54] and for its total flavonoids [55]. Its flavonoids and chalcones both demonstrate antioxidant activity [56]. Antitumour activities have been reported *in vitro *for both matrine and oxymatrine as well as for a number of flavonoids [57], of which trifolirhizin has shown both anti-inflammatory and antiproliferative actions [58], and kurarinone showed inhibition of immune response [59]. In human keratinocytes, an extract inhibited proinflammatory chemokines [60]. A *Sophora flavescens* extract showed antipruritic effects in a mouse model [61], and in a contact dermatitis mouse model, the topical application of a *Sophora flavescens* extract reduced hyperplasia, edema, spongiosis, and infiltration of mononuclear cells [51]. 

A series of studies have reported that the 70% ethanol extract, and its component momordin Ic, from the dried fruits of *Kochia scoparia *(Di fu zi) had antinociceptive and anti-inflammatory [62], antiallergic [63], and antipruritic effects [64]. An orally administered extract was found to be active in a rheumatoid arthritis model in rats [65].

Each of the above studies provides evidence that these four herbs have anti-inflammatory, antiproliferative and/or antipruritic activity, all of which are of relevance to the treatment of psoriasis. However, this evidence is indirect with only a few studies testing topical application. Consequently, whether the additional therapeutic effects of the CHM baths in the studies included in this review were produced by the specific effects of the CHMs and the effect of the warm water baths or were just nonspecific effects of an additional intervention remains unclear. Therefore, future studies should include a control group that uses a colored warm water bath plus phototherapy to investigate the specific efficacy of the CHMs.

### 4.3. Safety of Phototherapy Combined with CHM Bath for Psoriasis

Erythema and pruritus were the most common undesirable side effects of phototherapy reported by previous studies [18–21]. Consistently, some mild AEs related to phototherapy were reported by the ten studies included in this review. Three of them [5–7] concluded that the AEs in the treatment group were fewer than in the control group. However, none of the studies evaluated whether long-term use of a CHM bath could decrease the phototherapy dosage or frequency, and consequently reduce the AEs caused by phototherapy.

With regard to the frequently used CHMs, some AEs have been found for their oral use. Gastrointestinal reactions and headache have been reported following the use of *Salvia miltiorrhiza *root (Dan shen) [40] and liver toxicity has been associated with *Dictamnus dasycarpus *bark (Bai xian pi) [66]. However, no reports of AEs relating to the topical use of the four main herbs were located. Furthermore, no reports of phototoxicity associated with the oral or topical use of the four main herbs could be found. Therefore, these CHM baths appear to be safe for the management of psoriasis in the dosages used and over the short term. 

### 4.4. Outcome Measures

PASI 60 was suggested by “Consensus of Diagnosis and Treatment of Psoriasis Vulgaris in Integrative Medicine” in China [24], as a suitable threshold for effectiveness. Consequently, most of the included studies specified the numbers of participants who achieved PASI 60. However, this approach is not consistent with the treatment goals accepted internationally (PASI 75 or 50); this limits comparisons with international studies [25].

Moreover, three studies were excluded from meta-analysis since they used nonstandard scoring systems [11, 13, 16]. The inconsistency in effective rate calculations used in psoriasis studies was remarked upon in a recent review, which suggested unifying the diagnostic and efficacy evaluation standards for psoriasis [67]. In addition, since PASI score only focuses on lesion severity, other measurements such as BSA for assessing lesion size should be included in studies in order to provide a more complete assessment of overall severity.

### 4.5. Implications for Practice

CHM bath combined with phototherapy appears to be safe and may produce an additional benefit as an external treatment approach for psoriasis. An advantage of this method is it does not involve oral ingestion of medication. Its main limitations in clinical practice are the availability of bath facilities and the time required to prepare the bath. Based on the results of these studies, the four main herbs should be considered as key herbs when preparing a suitable bath formula.

### 4.6. Implications for Research

None of the studies mentioned blinding of participants, investigators, or outcome assessors; therefore, it could not be confirmed that the CHM baths were responsible for the additional effects or whether these were nonspecific effects due to the addition of an extra intervention. In future studies, a double-blind, placebo-controlled design should be conducted. Followup is also important in future studies to evaluate the long-term efficacy and safety of this therapy. Intention-to-treat analysis is necessary to reduce reporting bias. 

Although psoriasis itself is not a life-threatening condition, it has a significant impact on the sufferers' quality of life (QoL) [68, 69]. Quality of life is widely considered to be an important treatment goal [4, 25] but none of the thirteen studies investigated the effect on patients' QoL. Future studies should incorporate a QoL assessment such as the DLQI questionnaire. 

Psoriasis causes significant economic burden to patients and society [1, 4]. Phototherapy is generally provided in an outpatient department in China. This requires patients to travel to the hospital during working hours two or three times a week. Therefore, the cost-effectiveness of treatments should be assessed in future studies [4].

## 5. Conclusions

Chinese herbal medicine bath combined with phototherapy appears to show superior efficacy compared to phototherapy alone. However, there were methodological flaws in each of the included studies which could have led to bias. This combined therapy appears to be safe in the short term, but there was no long-time monitoring of efficacy and safety of these CHM baths combined with phototherapy. A number of the herbs used frequently in the baths have been evaluated in experimental studies and found to have actions of relevance to psoriasis treatment, so further investigation of these and other herbal bath ingredients is warranted.

## Figures and Tables

**Table 1:** Characteristics of thirteen studies of Chinese herbal medicine bath combined with phototherapy for psoriasis.

Author, year setting; location	Participant (T/C); average age (T/C)	Treatment interventions	Control interventions	Treatment and followup duration	Total treatment sessions	Outcome measures	Results	Number of participant reported adverse events (*n*=); SAE
Cui et al., 2008 [5] hospital; Beijing and Anshan, China	62/57; 45.82 ± 13.89/38.72 ± 12.17 years	CHM bath: 20 min, qod; NB-UVB: consistent with control intervention	NB-UVB: 50% of MED as initial dosage, increased by 0.1 J/cm^2^ each time, maximum dosage 2.5 J/cm^2^, twice a week	56 days; no information	16 sessions	TER based on PASI (90-60-25); PASI score	TER (T: 96.77%; C: 71.93%, *χ* ^2^ = 27.755, *P* < 0.01); PASI score (T: 4.16 ± 7.40; C: 11.40 ± 11.64); NB-UVB average dosage T: (9.95 ± 4.76) J/cm^2^; C: (12.77 ± 5.05) J/cm^2^ (*P* < 0.01)	Adverse events (redness, pruritus) rate: T: 4.84% (3/62); C: 31.58% (18/57) (*χ* ^2^ = 119, *P* < 0.01); no SAE

Gu et al., 2009 [6] hospital; Urumqi, China	89/96; 31.15/32.84 years	CHM bath: 30 min, qod; NB-UVB: consistent with control intervention; *Pu Lian* ointment, bid	NB-UVB (311 nm): 0.3 J/cm^2^ as initial dosage, increased by 0.1 J/cm^2^ each time, qod; *Pu Lian* ointment, bid	28 days; 6 months	14 sessions	TER based on PASI (90-60-25); relapse rate during followup	TER (T: 94.38%; C: 84.38%, *χ* ^2^ = 4.8, *P* < 0.05); relapse rate during followup (T: 10.81%; C: 30.30%, *P* < 0.05)	Mild redness, pruritus, and skin dryness (T: 10/89; C: 23/96) (*χ* ^2^ = 5.10, *P* < 0.05); skin pigmentation in all (resolved without medical assistance within 2 months); no SAE

Lin et al., 2010 [7] hospital; Hefei, China	95/90; 29.52 ± 6.38/ 27.42 ± 6.28 years	CHM bath: 20–30 min, three times a week; NB-UVB: consistent with control intervention; *Bing Huang Fu Le* ointment after NB-UVB, qd	NB-UVB (311 nm): 0.3–0.5 J/cm^2^ as initial dosage, increased by 0.1 J/cm^2 ^each time, 110 seconds, three times a week; *Bing Huang Fu Le* ointment after NB-UVB, qd	49 days; no information	20 sessions	TER based on PASI (90-60-25); PASI score	TER (T: 96.7%; C: 70%, *χ* ^2^ = 17.69, *P* < 0.01); PASI score (T: 11.15 ± 8.11; C: 14.74 ± 9.05)	Redness (T: 9/95; C: 22/90; *χ* ^2^ = 4.44, *P* < 0.05); pruritus (T: 11/95; C: 26/90; *χ* ^2^ = 4.94, *P* < 0.05); skin dryness (T: 15/95; C: 17/90; *P* > 0.05); no SAE

Liu et al., 2005 [8] hospital; Shijiazhuang, China	40/40; 36.2/35 years	CHM bath: 30 min, qod; NB-UVB: consistent with control intervention	NB-UVB (311–313 nm): 0.08 or 0.1 J/cm^2^ as initial dosage, increased by 0.01–0.03 J/cm^2^ each time, qod	40 days; no information	20 sessions	TER based on PASI (90-60-25)	TER (T: 95.0%; C: 82.5%, *P* < 0.01)	No information

Liu et al., 2004 [9] hospital; Dalian, China	151/179; no information	CHM bath: 20 min, qod; UVA and UVB: consistent with control intervention	UVA (5400 *μ*W/cm^2^): 0.16 J/cm^2^ as initial dosage, qod; UVB (350–460 *μ*W/cm^2^): 20.0 J/cm^2^ as initial, qod; increased by 10%–20% each time	No information; 12 months	No information	TER based on PASI (90-60-25); relapse rate during followup	TER (T: 90.07%; C: 88.83%, *P* > 0.05); relapse rate during followup (T: 15.23%; C: 35.19%, *P* < 0.05)	Redness, pruritus (T: 10/151; C: 10/179); no SAE

Shi et al., 2011 [10] hospital; Beijing, China	170/168; no information	CHM bath: 20 min, qod: NB-UVB: consistent with control intervention	NB-UVB (310–315 nm): 0.2–0.4 J/cm^2^ as initial dosage, increased by 0.1 J/cm^2 ^each time, qod	56 days; no information	28 sessions	TER based on PASI (90-60-20)	TER (T: 84.7%; C: 70.2%, *P* < 0.01)	Mild redness, pruritus (T: 11/170; C: 29/168); skin pigmentation in all (resolved without medical assistance within 3 months); no SAE

Wang et al., 2010 [11] hospital; Nanchong, China	70/70; no information	Acitretin capsules for one-week pretrial treatment: 20 mg/qd; CHM bath: 30 min, 3 times a week: NB-UVB: consistent with control intervention	Acitretin capsules: consistent with treatment intervention; NB-UVB: 0.5 J/cm^2^ as initial dosage, increased by 10%–20% each time, 3 times a week, or 1-2 times a week (if lesions reduced)	21 days; no information	9 sessions	TER based on lesion score (90-70-30)	TER (T: 77.14%; C: 58.57%, *χ* ^2^ = 5.534, *P* < 0.05)	Redness, pruritus (T: 6/70; C: 5/70); no SAE

Wang et al., 2011 [12] hospital; Kaifeng, China	50/50; 38.2/37.5 years	CHM bath: 30 min, qod; NB-UVB: consistent with control intervention; urea emollient after NB-UVB, qd	NB-UVB (310–315 nm): 0.3–0.5 J/cm^2 ^as initial dosage, increased by 10%–20% each time, qod; urea emollient after NB-UVB, qd	28 days; no information	14 sessions	TER based on PASI (90-60-20)	TER (T: 86.0%; C: 72.0%, *P* < 0.05)	Mild skin dryness, burning pain after NB-UVB (T: 4/50; C: 5/50); no SAE

Wang et al., 2012 [13] hospital; Kaifeng, China	60/60; 38.3/37.1 years	CHM bath: 30 min, qod; NB-UVB: consistent with control intervention; urea emollient after NB-UVB, qod; glycyrrhizin tablets: 2 tablets/tid	NB-UVB (310–315 nm): 0.3–0.5 J/cm^2^ as initial dosage, increased by 10%–20% each time, qod; urea emollient after NB-UVB, qod; glycyrrhizin tablets, 2 tablets/tid	28 days; no information	14 sessions	TER	TER (T: 91.7%; C: 77.7%, *χ* ^2^ = 8.239, *P* < 0.05)	Skin dryness, burning pain after NB-UVB (T: 4/60; C: 5/60); no SAE

Wu et al., 2011 [14] hospital; Chengdu, China	75/65; 32.5/34.8 years	Halometasone emollient for one-week pre-trial treatment: qd; CHM bath: 15–20 min, qod; NB-UVB: consistent with control intervention; urea emollient, qd	Halometasone emollient: consistent with treatment intervention; NB-UVB (310–315 nm): 0.5–0.6 J/cm^2^ as initial dosage, increased by 0.1 J/cm^2^ each time, qod; urea emollient, qd	40 days; no information	20 sessions	TER based on PASI (95-60-20); PASI score	TER (T: 78.67%; C: 56.92%, *χ* ^2^ = 10.54, *P* < 0.01); PASI score (T: 9.24 ± 2.17; C: 5.46 ± 1.86)	Slight light skin dryness, burning pain after NB-UVB (T: 2/75; C: 3/65); no SAE

Wu et al., 2010 [15] hospital; Guangzhou, China	40/39; 36.45 ± 8.52/36.67 ± 8.36 years	CHM bath: 15–20 min, qod; NB-UVB: consistent with control intervention	NB-UVB (311–315 nm): 0.3–0.5 J/cm^2^ as initial dosage, increased by 0.1 J/cm^2 ^each time, qod	90 days; no information	45 sessions	TER based on PASI (90-60-30); PASI score	TER (T: 75.00%; C: 51.28%, *χ* ^2^ = 4.78, *P* = 0.029); PASI score (T: 4.21 ± 1.22; C: 6.45 ± 2.27)	Skin dryness, pruritus (T: 5/40; C: 5/39); skin pigmentation in all; no SAE

Yan and Zhang, 2009 [16] hospital; Xi'an, China	42/38; no information	CHM bath: 30 min, qod; NB-UVB: consistent with control intervention	NB-UVB: 0.3 J/cm^2^ as initial dosage, increased by 0.2 J/cm^2^, qod	80 days; no information	40 sessions	TER based on lesion elimination	TER (T: 88.0%; C: 42.1%, *P* < 0.05)	No information

Zhou and Huang, 2005 [17] hospital; Nanning, China	143/129; 35/36.2 years	CHM bath: 30 min, qod; UVA: consistent with control intervention	UVA: 4 J/cm^2^ as initial dosage, increased by 1 J/cm^2^, qod	28 days; no information	14 sessions	TER based on PASI (90-60-25)	TER (T: 80.42%; C: 64.0%, *P* < 0.01)	No information

Qd: once a day, bid: twice a day, tid: three times a day, and qod: once in every two days; CHM: Chinese herbal medicine; NB-UVB: narrowband ultraviolet B; UVA: ultraviolet A; MED: minimal erythema dose, the minimum dose of radiation that produces skin erythema; TER: total effective rate, calculated as the percentage of “cured and remarkably effective” cases; PASI (95-60-20): “Clinically cured”-PASI score reduction of 95 to 100%; “remarkably effective”-PASI score reduction of 60 to 94%; “effective”-PASI score reduction of 20 to 59%; and “ineffective”-PASI score reduction of 0 to 19%; PASI (90-60-20), PASI (90-60-25), and PASI (90-60-30) refer to effectiveness levels using different proportions of PASI score reduction; lesion score (90-70-30): effectiveness levels based on the reduction of psoriatic lesions area and severity; lesion elimination: effectiveness levels based on the elimination of lesion (90–100%, 30–59%, or 0–29% refer to clinically cured, remarkably effective, and ineffective accordingly); SAE: serious adverse event.

**Table 2:** Chinese herbal medicine bath ingredients used in the thirteen studies.

Author, year (reference)	Chinese herbal medicine bath formula
Cui et al., 2008 [5]	*Galla Chinensis *(Wu bei zi)*, Cortex Phellodendri Chinensis *(Huang bo)*, Radix Angelica Sinensis *(Dang gui)*, Rhizoma Curcumae Longae *(Jiang huang),* Fructus Psoraleae* (Bu gu zhi), (no information of dosage)

Gu et al., 2009 [6]	*Radix et Rhizoma Salviae Miltiorrhizae *(Dan shen) 30 g, *Radix Angelica Sinensis *(Dang gui), *Spica Prunellae *(Xia ku cao) 30 g, *Fructus Kochiae *(Di fu zi) 30 g, *Cortex Dictamni *(Bai xian pi) 30 g, *Cortex Phellodendri Chinensis *(Huang bo) 30 g, *Folium Isatidis *(Da qing ye) 30 g, *Rhizoma Smilacis Glabrae *(Tu fu ling) 30 g

Lin et al., 2010 [7]	*Rhizoma Smilacis Glabrae *(Tu fu ling) 30 g, *Fructus Kochiae *(Di fu zi) 15 g, *Radix Angelica Sinensis *(Dang gui) 20 g, *Cortex Dictamni *(Bai xian pi) 20 g, *Radix et Rhizoma Cynanchi Paniculati *(Xu chang qing) 20 g, *Radix Sophora Flavescens *(Ku shen) 15 g, *Fructus Cnidii *(She chuang zi) 20 g, *Fructus Xanthii *(Cang er zi) 15 g, *Radix Stemonae *(Bai bu) 15 g, *Cortex Pseudolaricis *(Tu jing pi) 10 g, *Fructus Tribuli *(Ji li) 15 g, *Radix et Rhizoma Rhei *(Da huang) 10 g

Liu et al., 2005 [8]	*Radix et Rhizoma Cynanchi Paniculati *(Xu chang qing) 30 g, *Fructus Cnidii *(She chuang zi) 30 g, *Radix Sophora Flavescens *(Ku shen) 30 g, *Pericarpium Zanthoxyli *(Hua jiao) 30 g, *Herba cum Radice Patriniae *(Bai jiang cao) 30 g, *Rhizoma et Radix Polygoni Cuspidati *(Hu zhang) 30 g, *Herba Portulacae *(Ma chi xian) 30 g, *Radix et Rhizoma Salviae Miltiorrhizae *(Dan shen) 20 g, *Rhizoma Atractylodis *(Cang zhu) 15 g

Liu et al., 2004 [9]	Blood-heat syndrome: *Folium Isatidis *(Da qing ye)*, Radix Rehmanniae *(Di huang),* Radix Paeoniae Rubra *(Chi shao),* Rhizoma Smilacis Glabrae *(Tu fu ling)*, Radix Arnebiae *(Zi cao),* Cortex Moutan *(Mu dan pi); Blood-stasis syndrome: *Radix et Rhizoma Salviae Miltiorrhizae *(Dan shen),* Semen Persicae *(Tao ren)*, Flos Carthami *(Hong hua),* Rhizoma Curcumae *(E zhu),* Caulis Spatholobi *(Ji xue teng); Blood-dryness syndrome: *Radix Rehmanniae *(Di huang)*, Radix Angelica Sinensis *(Dang gui),* Cortex Dictamni *(Bai xian pi)*, Herba Hedyotis *(Bai hua she she cao),* Rhizoma Smilacis Glabrae *(Tu fu ling), (no information of dosage)

Shi et al., 2011 [10]	*Caulis Impatientis *(Tou gu cao),* Cacumen Platycladi *(Ce bai ye),* Cortex Dictamni *(Bai xian pi)*, Radix et Rhizoma Salviae Miltiorrhizae *(Dan shen),* Radix Angelica Sinensis *(Dang gui), *Semen Persicae *(Tao ren)*, Radix Sophora Flavescens *(Ku shen)*, Fructus Kochiae *(Di fu zi)*, Margarita *(Zhen zhu), (no information of dosage)

Wang et al., 2010 [11]	*Flos Chrysanthemi Indici *(Ye ju hua) 240 g, *Pericarpium Zanthoxyli *(Hua jiao) 120 g, *Rhizoma Smilacis Glabrae *(Tu fu ling) 150 g, *Radix Sophora Flavescens *(Ku shen) 300 g, *Fructus Kochiae *(Di fu zi) 300 g

Wang et al., 2011 [12]	*Radix Scutellariae *(Huang qin),* Herba cum Radice Patriniae *(Bai jiang cao)*, Herba Taraxaci *(Pu gong ying)*, Radix Paeoniae Rubra *(Chi shao)*, Cortex Moutan *(Mu dan pi)*, Radix et Rhizoma Salviae Miltiorrhizae *(Dan shen),* Radix Asparagi *(Tian dong)*, Radix Rehmanniae *(Di huang)*, Cortex Dictamni *(Bai xian pi)*, Fructus Kochiae *(Di fu zi)*, Rhizoma Atractylodis *(Cang zhu), (no information of dosage)

Wang et al., 2012 [13]	*Radix Scutellariae *(Huang qin)*, Herba cum Radice Patriniae *(Bai jiang cao)*, Herba Taraxaci *(Pu gong ying)*, Radix Paeoniae Rubra *(Chi shao)*, Cortex Moutan *(Mu dan pi)*, Radix et Rhizoma Salviae Miltiorrhizae *(Dan shen),* Radix Asparagi *(Tian dong)*, Radix Rehmanniae *(Di huang)*, Cortex Dictamni *(Bai xian pi)*, Fructus Kochiae *(Di fu zi)*, Rhizoma Atractylodis *(Cang zhu), (no information of dosage)

Wu et al., 2011 [14]	*Radix Sophora Flavescens *(Ku shen), *Radix et Rhizoma Cynanchi Paniculati *(Xu chang qing), *Flos Chrysanthemi Indici *(Ye ju hua), *Herba Violae *(Zi hua di ding), *Flos Lonicerae Japonicae *(Jin yin hua), *Fructus Cnidii *(She chuang zi), *Glycyrrhiza uralensis *root, (no information of dosage)

Wu et al., 2010 [15]	*Radix Rehmanniae *(Di huang) 40 g, *Flos Lonicerae Japonicae *(Jin yin hua)* *30 g, *Herba Violae *(Zi hua di ding) 30 g, *Herba Taraxaci *(Pu gong ying)* *30 g, *Zaocys *(Wu shao she) 15 g, *Radix Scutellariae *(Huang qin) 30 g, *Radix Arnebiae seu Lithospermi *(Zi cao) 30 g

Yan and Zhang, 2009 [16]	*Radix Sophora Flavescens *(Ku shen) 30 g, *Fructus Cnidii *(She chuang zi) 30 g, *Radix Clematidis *(Wei ling xian) 30 g, *Cortex Dictamni *(Bai xian pi) 30 g, *Radix et Rhizoma Salviae Miltiorrhizae *(Dan shen) 20 g, *Rhizoma Atractylodis *(Cang zhu)* *20 g, *Rhizoma Smilacis Glabrae *(Tu fu ling) 20 g

Zhou and Huang, 2005 [17]	*Rhizoma Coptidis *(Huang lian) 10 g, *Cortex Phellodendri Chinensis *(Huang bo) 20 g,* Radix Sophora Flavescens *(Ku shen)* *20 g, *Radix Sanguisorbae *(Di yu) 20 g, *Cortex Mahonia bealei *(Tu Huang bo) 60 g, *Pericarpium Granati *(Shi liu pi) 15 g, *Fructus Kochiae *(Di fu zi) 15 g, *Herba Senecionis Scandens *(Qian li guang) 30 g
